# Signaling Pathways Linked to Serotonin-Induced Superoxide Anion Production: A Physiological Role for Mitochondria in Pulmonary Arteries

**DOI:** 10.3389/fphys.2017.00076

**Published:** 2017-02-09

**Authors:** Nafiisha Genet, Marie Billaud, Rodrigue Rossignol, Mathilde Dubois, Jennifer Gillibert-Duplantier, Brant E. Isakson, Roger Marthan, Jean-Pierre Savineau, Christelle Guibert

**Affiliations:** ^1^Centre de Recherche Cardio-Thoracique de Bordeaux, Institut National de la Santé et de la Recherche Médicale (INSERM), U1045Bordeaux, France; ^2^Centre de Recherche Cardio-Thoracique de Bordeaux, Université de BordeauxBordeaux, France; ^3^Robert M. Berne Cardiovascular Research CenterCharlottesville, VA, USA; ^4^Maladies Rares: Génétique et Métabolisme, Université de BordeauxBordeaux, France

**Keywords:** superoxide anion, 5-HT, mitochondria, pulmonary artery, calcium

## Abstract

Serotonin (5-HT) is a potent vasoconstrictor agonist and contributes to several vascular diseases including systemic or pulmonary hypertension and atherosclerosis. Although superoxide anion (${{\text{O}}_{2}}^{\underline{•}}$) is commonly associated to cellular damages due to ${{\text{O}}_{2}}^{\underline{•}}$ overproduction, we previously demonstrated that, in physiological conditions, ${{\text{O}}_{2}}^{\underline{•}}$ also participates to the 5-HT contraction in intrapulmonary arteries (IPA). Here, we focused on the signaling pathways leading to ${{\text{O}}_{2}}^{\underline{•}}$ production in response to 5-HT in rat IPA. Using electron paramagnetic resonance on rat IPA, we showed that 5-HT (100 μM)-induced ${{\text{O}}_{2}}^{\underline{•}}$ production was inhibited by ketanserin (1 μM—an inhibitor of the 5-HT_2_ receptor), absence of extracellular calcium, two blockers of voltage-independent calcium permeable channels (RHC80267 50 μM and LOE-908 10 μM) and a blocker of the mitochondrial complex I (rotenone—100 nM). Depletion of calcium from the sarcoplasmic reticulum or nicardipine (1 μM—an inhibitor of the L-type voltage-dependent calcium channel) had no effect on the 5-HT-induced ${{\text{O}}_{2}}^{\underline{•}}$ production. ${{\text{O}}_{2}}^{\underline{•}}$ levels were also increased by α-methyl-5-HT (10 μM—a 5-HT_2_ receptors agonist) whereas GR127935 (1 μM—an antagonist of the 5-HT_1B/D_ receptor) and citalopram (1 μM—a 5-HT transporter inhibitor) had no effect on the 5-HT-induced ${{\text{O}}_{2}}^{\underline{•}}$ production. Peroxynitrites were increased in response to 5-HT (100 μM). In isolated pulmonary arterial smooth muscle cells loaded with rhod-2 or mitosox probes, we respectively showed that 5-HT increased both mitochondrial calcium and ${{\text{O}}_{2}}^{\underline{•}}$ levels, which were both abrogated in absence of extracellular calcium. Mitochondrial ${{\text{O}}_{2}}^{\underline{•}}$ levels were also abolished in the presence of rotenone (100 nM). In pulmonary arterial smooth muscle cells loaded with TMRM, we showed that 5-HT transiently depolarized the mitochondrial membrane whereas in the absence of extracellular calcium the mitochondrial membrane depolarisation was delayed and sustained in response to 5-HT. 5-HT decreased the mitochondrial respiratory rate measured with a Clark oxygen electrode. Altogether, in physiological conditions, 5-HT acts on 5-HT_2_ receptors and induces an ${{\text{O}}_{2}}^{\underline{•}}$ production dependent on extracellular calcium and mitochondria.

## Introduction

Several agonists acting on seven transmembrane domain receptors (G-protein coupled receptors) are involved in vascular tone. Serotonin (5-HT) is a potent vasoconstrictor agonist under physiological conditions and contributes to several vascular diseases (namely systemic or pulmonary hypertension and atherosclerosis). In the lung, 5-HT is locally released by pulmonary neuroendocrine cells, endothelial cells and neuroepithelial bodies distributed throughout the airways (Maclean and Dempsie, 2010). In the cardiovascular system, 5-HT has a potent mitogenic and contractile effect and its concentration is increased in pulmonary hypertension (Kéreveur et al., 2000; Guibert et al., 2004; Rodat et al., 2007; Rodat-Despoix et al., 2008). The mitogenic effect of 5-HT in the pulmonary artery (PA) is dependent on 5-HT uptake through the 5-HT transporter (Maclean and Dempsie, 2010) and the activation of 5-HT receptors whereas the contractile effect of 5-HT is mainly due to its action on 5-HT_2A_ and 5-HT_1B/D_ receptors (Rodat-Despoix et al., 2008).

We previously demonstrated, in rat intrapulmonary arteries (IPA), that 5-HT increases superoxide anion (${{\text{O}}_{2}}^{\underline{•}}$) levels in smooth muscle and such ${{\text{O}}_{2}}^{\underline{•}}$ participates to pulmonary vasoconstriction (Billaud et al., 2009). Nevertheless, so far, no studies have yet elucidated the signaling pathways that are triggered in this process.

5-HT is also well-known to stimulate reactive oxygen species (ROS) production in human, bovine, and mice pulmonary artery smooth muscle cells (PASMC) and the production of ${{\text{O}}_{2}}^{\underline{•}}$ facilitates 5-HT-induced pulmonary vasoconstriction as well as smooth muscle cells proliferation (Lee et al., 1998, 1999, 2001; Liu and Folz, 2004; Lawrie et al., 2005; Peña-Silva et al., 2009). While the role of ROS in 5-HT-induced PASMC proliferation has been extensively studied, only one study, on mouse PA, addressed the role of ROS in 5-HT-induced contraction (Liu and Folz, 2004). Indeed, Liu and Folz showed that 5-HT enhances ${{\text{O}}_{2}}^{\underline{•}}$ levels via NADPH oxidase stimulation in the smooth muscle (Liu and Folz, 2004). This increased ${{\text{O}}_{2}}^{\underline{•}}$ was localized in the extracellular space and further increased the contraction to 5-HT. However, so far, the precise mechanisms involved in the enhanced 5-HT-induced contraction by ${{\text{O}}_{2}}^{\underline{•}}$ in mice PA have not been addressed to date and the exact role of 5-HT_2_ receptors, 5-HT transporter and mitochondria (another important source of ${{\text{O}}_{2}}^{\underline{•}}$) still remain unknown (Cogolludo et al., 2006; Perez-Vizcaino et al., 2010).

In human as in rats, PA contraction to 5-HT is dependent on myofilament Ca^2+^-sensitization and cytosolic calcium concentration (Guibert et al., 2004; Rodat-Despoix et al., 2008, 2009). Intracellular calcium level is modulated by the activity of calcium permeable channels localized at the plasma membrane and in the sarcoplasmic reticulum. Upon binding to its 5-HT_2_ receptors, 5-HT induces a rise in cytosolic calcium coming from both (i) the intracellular compartment, mainly the sarcoplasmic reticulum, and (ii) an influx of extracellular calcium (Guibert et al., 2004). In pulmonary arterial smooth muscle, calcium influx is due to the activation of voltage-dependent calcium channels and voltage-independent calcium channels (Guibert et al., 2004; Ducret et al., 2008; Rodat-Despoix et al., 2008). However, we have previously shown, in rat IPA, that both the contraction and the calcium response to 5-HT are strongly dependent on voltage-independent calcium channels compared to voltage-dependent calcium channels (Guibert et al., 2004; Rodat et al., 2007; Rodat-Despoix et al., 2008).

Altogether, since the mitochondrial electron transport chain (METC) is known to be sensitive to calcium to produce ${{\text{O}}_{2}}^{\underline{•}}$ (Denton et al., 1978; Archer et al., 1993; Yuan et al., 1993; Leach et al., 2001; Dromparis and Michelakis, 2013; Freund-Michel et al., 2014; Yumnam et al., 2016) we have investigated, in the present study, the signaling pathways involved in 5-HT-induced ${{\text{O}}_{2}}^{\underline{•}}$ increase in rat IPA by focusing on the mitochondria as an important source of ROS and the role of intra and extracellular calcium.

## Materials and methods

### Tissue preparation

Male Wistar rats (weighing 300–400 g) were sacrificed using an intraperitoneal injection of pentobarbital (150 mg/kg) according to the animal care and use local committee (Comité d'éthique régional d'Aquitaine). All the experiments were carried out in accordance with the recommendations of the Comité d'éthique régional d'Aquitaine (CEEA 50) and the protocol was approved by the same committee (protocol n° 50110016-A). The left lung was rapidly removed and rinsed in Krebs–HEPES–bicarbonate (KHB) containing (in mM): 118.4 NaCl, 4.7 KCl, 1.2 MgSO_4_, 4 NaHCO_3_, 1.2 KH2PO_4_, 2 CaCl_2_, 10 N-2-hydroxyethylpiperazine-N'-2-ethanesulfonic acid (HEPES) and 6 D-glucose, pH 7.4 with NaOH. Intrapulmonary arteries (IPA) of first, second and third order with an external diameter ranging from 300 μm to 2 mm were then dissected free from surrounding connective tissues under binocular control.

### Electronic paramagnetic resonance (EPR) recordings

EPR recordings were performed as previously described (Billaud et al., 2009). IPA were incubated in the spin trap solution containing 500 μM 1-hydroxy-3-methoxycarbonyl-2,2,5,5-tetramethylpyrrolidin (CMH, Noxygen), 25 μM deferoxamine (Sigma), and 5 mM N,N-diethyldithiocarbamate (DETC, Sigma) in KHB at 37°C for 45 min. 5-HT was added during the spin trap incubation. When indicated, pharmacological inhibitors were also added 30 min prior and during the spin trap incubation. The reaction was stopped by snap freezing the samples in liquid nitrogen. Samples were then analyzed by EPR spectrometry using a tabletop X-band spectrometer miniscope (MS200, Magnettech). Spectra of the oxidized product of CMH (CM^•^) were recorded at 77°K using a flask Dewar. Acquisition parameters were as followed: Bo Field: 3341 ± 150 G, microwave power: 10 dB, amplitude modulation: 5 G, sweep time: 60 s, gain: 300 and 3 scans. Signals were quantified by measuring the total amplitude of the signal, after correction of baseline and normalized to the protein quantity of the sample in mg/ml. ${{\text{O}}_{2}}^{\underline{•}}$ level was expressed as [A/(mg/ml) of proteins], where A corresponds to arbitrary units.

### Quantification of peroxynitrites

IPA were incubated with or without 5-HT 100 μM in phosphate buffer saline solution during 45 min. Arteries were then homogenized in this incubation medium and centrifuged (10500 g, 20 min at 4°C). Supernatants were further used for the measurement of peroxynitrites. As peroxynitrites are rapidly transformed to their more stable structural isomer, nitrates, supernatants were first incubated with nitrate reductase (Cayman chemicals) to convert nitrates into nitrites. Levels of peroxynitrites were then quantified from nitrites with Griess reagent according to the manufacturer's instructions (Molecular probes). Peroxynitrites levels were normalized to tissue protein content in mg/ml. Results are expressed as [(μM)/(mg/ml) of proteins].

### Measurement of superoxide dismutase (SOD) activity

IPA treated or not with 5-HT were homogenized in HEPES buffer containing (in mM): 20 HEPES, 1 EGTA, 210 mannitol, 70 sucrose, pH 7.2 with NaOH. Homogenized samples were centrifuged (1500 g, 5 min at 4°C) and supernatants were used for the determination of superoxide dismutase (SOD) activity. SOD enzyme activity was then determined as described in the procedure of the SOD Assay Kit-WST from Sigma and as carried out in previous studies (Peskin and Winterbourn, 2000). Briefly, the method is based on the inhibition of the SOD activity in presence of the highly water-soluble tetrazolium salt WST-1 [2-(4-Iodophenyl)-3-(4-nitrophenyl)-5-(2,4-disulfophenyl)-2H-tetrazolium, monosodium salt] that produces a colored water-soluble formazan dye (WST1-formazan) upon reduction with ${{\text{O}}_{2}}^{\underline{•}}$. Levels of SOD activity were determined by measuring the absorbance of the WST1-formazan dye at 450 nm with a microplate reader (EL808, Bio-Tek instruments). SOD standard solutions ranging from 0.001 to 200 Units/ml were used to perform a calibration curve and SOD activity value was read from this curve and expressed as Units/ml for each sample. Tissue protein content of each sample was also quantified in mg/ml to normalize SOD enzyme activity, expressed as Units/mg of proteins.

### Quantification of total protein levels

Briefly, IPA were lysed in RIPA buffer (Sigma) for total protein extraction. The lysed tissues were centrifuged at 15,000 g for 10 min at 4°C. The amount of total protein in the supernatant was measured using a lowry assay (Biorad DC protein assay) following the manufacturer's instructions. The amount of protein in each sample was compared to a standard curve performed with bovine serum albumin (BSA, 0–2 mg/ml).

### Pulmonary arterial smooth muscle cells culture

PASMC were obtained as previously described (Martin et al., 2012). Briefly, IPA from the rat left lung were dissected free from surrounding connective tissues. IPA were cut into small pieces and placed in Hanks Balanced Salt Solution containing 50 μM CaCl_2_, 0.5 mg/ml papain, 0.3 mM dithioerythritol, and 0.3 mg/ml collagenase for 10 min at 37°C. IPA were then mechanically and gently agitated using a polished wide-bore Pasteur pipette to release the cells. PASMC were seeded onto 14 mm round glass coverslips, maintained in culture medium (DMEM) supplemented with 1% penicillin– streptomycin, 1% sodium pyruvate, 1% non essential amino acids, and 10% fetal calf serum. Cells were stored at 37°C in a humidified atmosphere gassed with 5% CO_2_. When the PASMC reached 80% confluence, they were growth arrested by using serum-free culture medium supplemented with 1% insulin–transferrin–selenium. After 48 h, recordings of cytosolic and mitochondrial calcium, mitochondrial membrane potential, and mitochondrial ${{\text{O}}_{2}}^{\underline{•}}$ measurements with fluorescent dyes were performed. For mitochondrial respiration assessment, PASMC were seeded in 175-cm^2^ flasks and grown until confluence. Cells were used up to passage 4. PASMC phenotype was confirmed by positive immunostaining for α-smooth muscle actin and calponin.

### Measurements of mitochondrial respiration

Endogenous cellular oxygen consumption was monitored on a suspension of PASMC at 37°C in a 1 ml thermostatically controlled chamber (1.0 × 10^6^ cells/ml/run) equipped with a Clark oxygen electrode (Oxygraph system, Hansatech) as previously described (Rossignol et al., 2004). The respiratory buffer was the DMEM growth medium without serum. Cumulative concentrations of 5-HT were added to the chamber when indicated. The basal respiratory rate is expressed as nmol O_2_/min/10^6^ cells and the effect of 5-HT was calculated as a percentage of the basal respiratory rate.

### Recording of cytosolic and mitochondrial calcium and mitochondrial membrane potential

Cytosolic and mitochondrial calcium were assessed by simultaneously loading PASMC with the non ratiometric fluo4-AM probe (2 μM) and rhod-2AM probe (1 μM) respectively. PASMC were also loaded simultaneously with rhod-2AM (1 μM) and mitotracker green (400 nM), a mitochondrial marker, for mitochondria labeling. For mitochondrial membrane potential measurements, PASMC were loaded with tetramethylrhodamine methyl ester (TMRM) (100 nM). All the fluorescent dyes used were obtained from Molecular probes (Invitrogen). For all the above mentioned experiments, PASMC were loaded with the dyes for 30 min at 37°C in KHB solution and then washed in KHB solution without the dye for 30 min to allow deesterification of the dye. PASMC were then mounted on the stage of a laser scanning confocal microscope (TE2000, Nikon, Champigny-Sur-Marne, France) with a x 20, 1.40 NA plan apochromat oil-immersion objective. The cells were under continuous bath perfusion with KHB solution at a rate of 2 ml/min and 5-HT 100 μM was bath-applied. Fluo-4AM was excited with an argon laser at 488 nm and emitted light was filtered at 515 ± 30 nm. Rhod-2AM and TMRM were excited with a helium-neon laser at 543 nm and the emitted light was filtered at 605 ± 75 nm. Regions of interest were drawn around each cell and fluorescence was recorded every 2 s by using the EZ-C1 software (Nikon, Champigny-Sur-Marne, France). All experiments were performed at room temperature. The fluorescence values (F) were normalized to the basal fluorescence (fluorescence before application of the agonists or F0) to obtain the fluorescence ratio (F/F0). The area under the curve was calculated with Origin 6.0 software (Microcal). The area under the curve was calculated for a 75 s duration, starting 45 s after the beginning of the recording, which corresponds to the time where the agonist was applied. Results are expressed as (F/F0)s^−1^.

### Measurement of mitochondrial ${{\text{O}}_{2}}^{\underline{•}}$ production

Mitochondrial ${{\text{O}}_{2}}^{\underline{•}}$ production was measured with mitoSox red (molecular probes). PASMC were loaded with mitoSox red (4 μM) in KHB for 20 min at 37°C. PASMC were washed twice and mounted on the stage of a laser scanning confocal microscope (TE2000, Nikon, Champigny-Sur-Marne, France) with a x 20, 1.40 NA plan apochromat oil-immersion objective. 5-HT (100 μM) was applied at *t* = 0 min. MitoSox red was excited with an helium-neon laser at 543 nm and the emitted light was filtered at 605 ± 75 nm. Regions of interest were drawn around each cell and fluorescence was recorded every 15 min for 60 min by using the EZ-C1 software (Nikon, Champigny-Sur-Marne, France). The fluorescence values at the start of the experiment (F0) were subtracted from the fluorescence at *t* = 60 min (F) to calculate the percentage of increase in mitoSox red fluorescence.

### Drugs and chemical reagents

All drugs were diluted in distilled water, except for LOE-908, nicardipine, RHC-80267, and rotenone, which were dissolved in dimethyl sulphoxide (DMSO). Antimycin A was dissolved in ethanol. The maximal concentration of DMSO and ethanol used in experiments was <0.1%.

### Data analysis and statistics

All results are expressed as mean ± SEM; *n* indicates the number of rats for EPR, peroxynitrites measurements, SOD activity and mitochondrial respiratory rate, the number of cells for cytosolic, mitochondrial calcium, mitochondrial membrane potential, and mitochondrial superoxide anion recordings. Experiments were performed on a minimum of 3–4 rats. Unpaired *t*-test was used to compare data obtained from measurements of cytosolic, mitochondrial calcium levels, and mitochondrial membrane potential. Non-parametric one way ANOVA (Kruskal-Wallis) followed by Dunn's multiple comparison tests was used to compare mitochondrial respiration. All other data were analyzed using a non-parametric test for unpaired samples (Mann-Whitney test). All bar graphs and statistics were performed with GraphPad Prism 5. Values of *P* < *0.05* were considered significant.

## Results

### 5-HT-induced production of ${{\text{O}}_{2}}^{\underline{•}}$ in rat IPA

**Figure 1:**
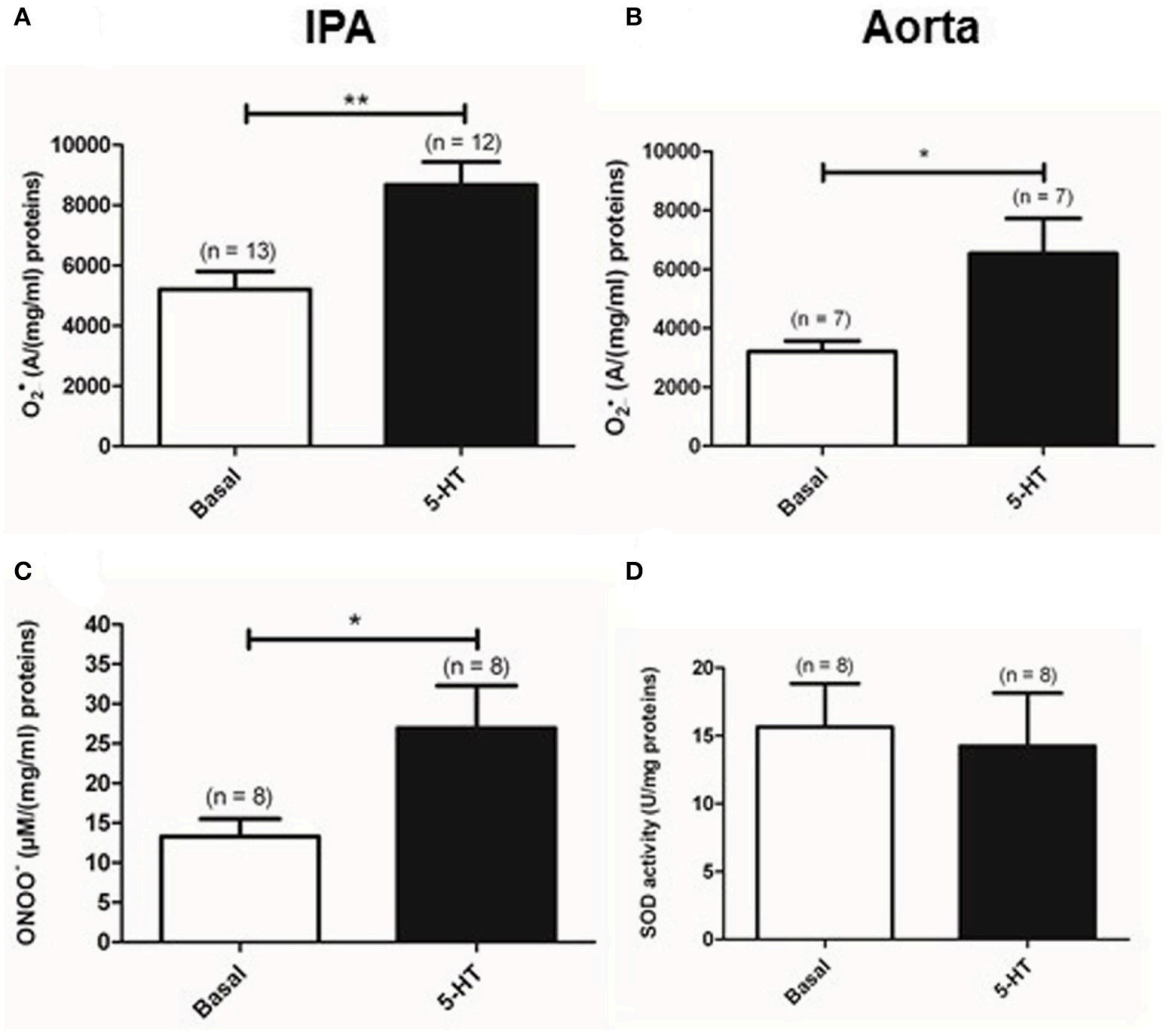
**Production of ${{\text{O}}_{2}}^{\underline{•}}$ by 100 μM 5-HT**. Levels of ${{\text{O}}_{2}}^{\underline{•}}$ were measured by EPR spectrometry in basal conditions (white column) or in the presence of 5-HT (black column) in rat IPA **(A)** or aorta **(B)**. **(C)** 5-HT increases ONOO^−^ levels in rat IPA. **(D)** 5-HT does not modify SOD activity measured in rat IPA. Data are expressed as mean ± SEM. ^**^ and ^*^ mean a significant difference for *P* < 0.01 and *P* < 0.05 respectively. n is the number of rats.

We previously demonstrated that 5-HT had a maximal contractile effect at the concentration of 100 μM in rat IPA (Billaud et al., 2009). Moreover, the maximal effect of the anti-oxidant PEG-SOD and PEG-catalase was observed on the contraction to 5-HT 100 μM (Billaud et al., 2009). Therefore, we focused our study on ${{\text{O}}_{2}}^{\underline{•}}$ produced by 5-HT 100 μM. Indeed, as previously demonstrated (Billaud et al., 2009), a new set of experiments has been performed with electron paramagnetic resonance (EPR) recordings confirming that 5-HT (100 μM) significantly increased ${{\text{O}}_{2}}^{\underline{•}}$ levels in rat IPA (*n* = 13 and 12) (Figure 1A). Similarly, 5-HT (100 μM) also significantly increased ${{\text{O}}_{2}}^{\underline{•}}$ levels in rat aorta (*n* = 7) (Figure 1B). Since ${{\text{O}}_{2}}^{\underline{•}}$ can be scavenged by NO to form peroxynitrites (ONOO^−^), we measured ONOO^−^ concentration in rat IPA with Griess reagent as an alternative means to investigate 5-HT-induced ${{\text{O}}_{2}}^{\underline{•}}$ increase. 5-HT significantly increased ONOO^−^ levels (Figure 1C). Moreover, in rat IPA, 5-HT had no significant effect on the activity of superoxide dismutase, an anti-oxidant enzyme responsible for ${{\text{O}}_{2}}^{\underline{•}}$ degradation (Figure 1D). Altogether, 5-HT produces ${{\text{O}}_{2}}^{\underline{•}}$ in IPA and aorta and modification of superoxide dismutase activity cannot explain 5-HT-induced ${{\text{O}}_{2}}^{\underline{•}}$ increase in IPA.

### Role of 5-HT receptors, 5-HT transporter and calcium in 5-HT-induced ${{\text{O}}_{2}}^{\underline{•}}$ increase

**Figure 2:**
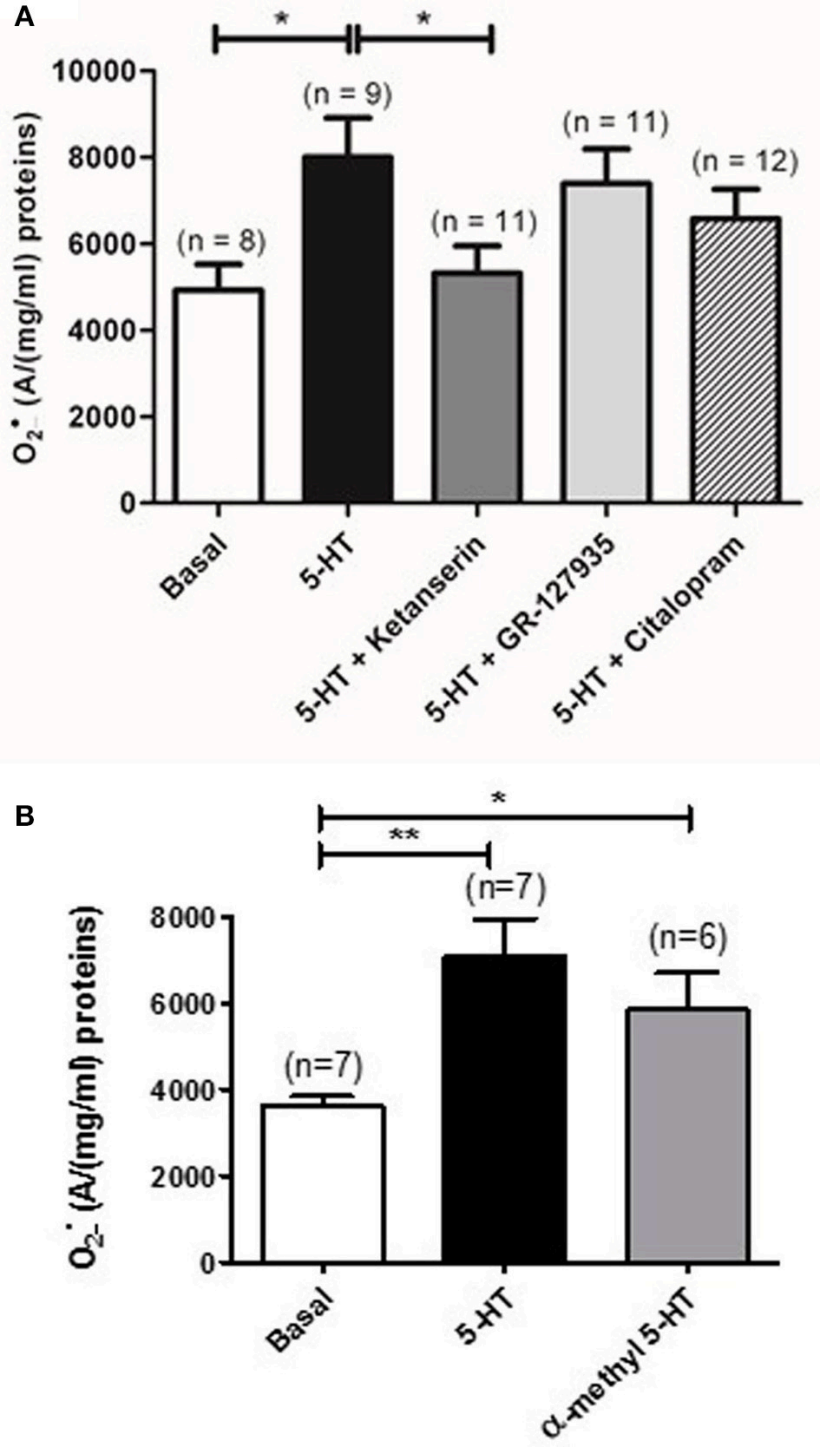
**Role of 5-HT receptors and 5-HT transporter in 5-HT-induced ${{\text{O}}_{2}}^{\underline{•}}$ increase in rat IPA**. **(A)** Effect of a 5-HT_2_ receptor antagonist (ketanserin, 1 μM, dark gray column), a 5-HT_1_ receptor antagonist (GR127935, 1 μM, light gray column) or a 5-HT transporter antagonist (citalopram, 1 μM, hatched column) on the ${{\text{O}}_{2}}^{\underline{•}}$ produced by 100 μM 5-HT. **(B)** α-methyl 5-HT 10 μM significantly increases ${{\text{O}}_{2}}^{\underline{•}}$ levels in rat IPA. Data are expressed as mean ± SEM. ^*^ and ^**^ indicate a significant difference with *P* < 0.05 and *P* < 0.01 respectively. n is the number of rats.

Pulmonary arterial contraction in response to 5-HT is known to be mediated by ${{\text{O}}_{2}}^{\underline{•}}$, 5-HT receptors (5-HT_2A_ and 5-HT_1B/D_) and, to a lesser extent, by the 5-HT transporter (Morecroft et al., 2005; Billaud et al., 2009). We have thus used selective pharmacological inhibitors and activators to study the role of the 5-HT transporter, 5-HT_2_, and 5-HT_1B/D_ receptors involved in IPA contraction. EPR recordings showed that ketanserin (1 μM), a 5-HT_2_ receptor antagonist significantly decreased the amount of ${{\text{O}}_{2}}^{\underline{•}}$ produced by 5-HT (*n* = 9) (Figure 2A) whereas blocking the 5-HT_1B/D_ receptors or the 5-HT transporter by GR 127935 (1 μM) or citalopram (1 μM), respectively, had no effect (*n* = 11–12) (Figure 2A). Moreover, α-methyl 5-HT (10 μM), a non-selective 5-HT_2_ agonist significantly increased ${{\text{O}}_{2}}^{\underline{•}}$ levels (*n* = 6) (Figure 2B).

**Figure 3:**
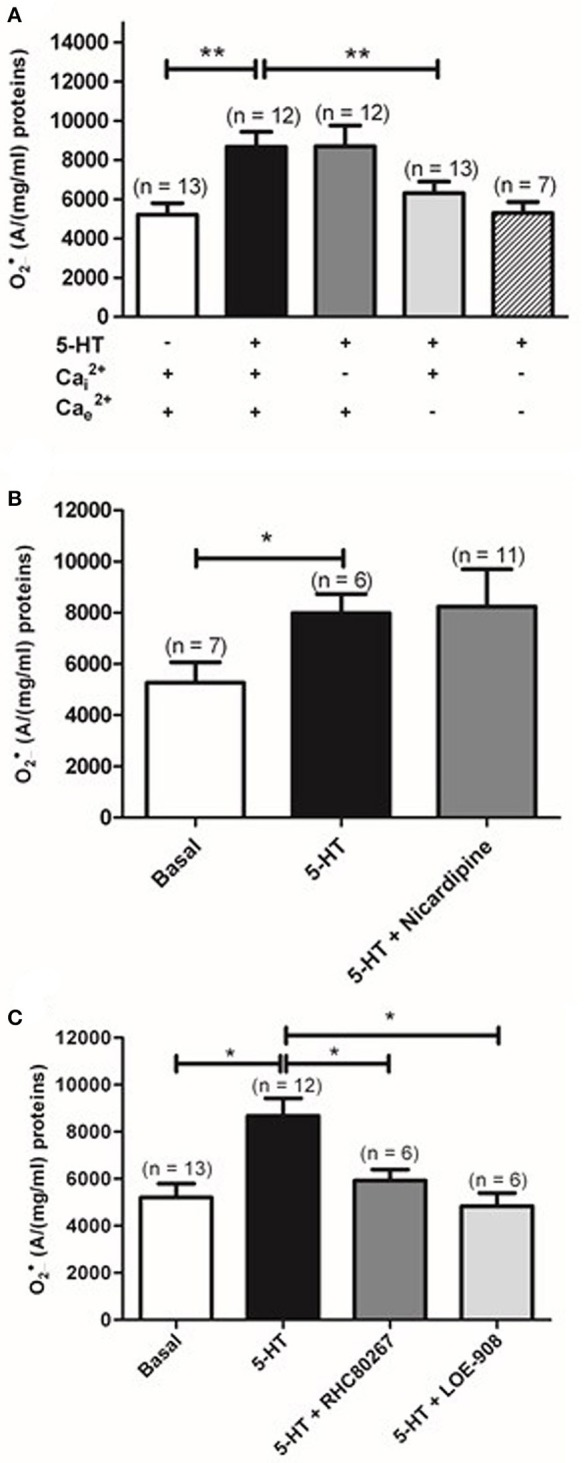
**Role of calcium and calcium permeable channels in 5-HT-induced ${{\text{O}}_{2}}^{\underline{•}}$ increase in rat IPA**. **(A)** Effect of removal of Ca^2+^ from the sarcoplasmic reticulum (${\text{Ca}}_{\text{i}}^{2+}$, dark gray column) or extracellular Ca^2+^ (${\text{Ca}}_{\text{e}}^{2+}$, light gray column), or both ${\text{Ca}}_{\text{i}}^{2+}$and ${\text{Ca}}_{\text{e}}^{2+}$ (hatched column) on ${{\text{O}}_{2}}^{\underline{•}}$ produced by 100 μM 5-HT and measured by EPR spectrometry. Effect of nicardipine (1 μM), an L-type voltage-gated calcium channel inhibitor (dark gray column) or two antagonists of voltage-independent calcium permeable channel, RHC-80267 (50 μM) and LOE-908 (10 μM) (dark and light gray columns respectively) on the ${{\text{O}}_{2}}^{\underline{•}}$ produced by 100 μM 5-HT (**B** and **C** respectively). Data are expressed as mean ± SEM. ^**^ and ^*^ mean a significant difference for *P* < 0.01 and *P* < 0.05 respectively. n is the number of rats.

Given the importance of calcium in the contraction to 5-HT in rat IPA, we have then studied the role of intracellular calcium by depleting the calcium from the sarcoplasmic reticulum with a calcium free KHB solution containing a calcium chelator (0.4 mM EGTA), an activator of ryanodine receptors (caffeine 5 mM) and an inhibitor of the sarcoplasmic reticulum Ca^2+^-ATPase (thapsigargin 1 μM) to prevent calcium re-uptake in the sarcoplasmic reticulum. Such protocol has been shown to fully deplete sarcoplasmic reticulum in calcium in rat pulmonary arteries (Gonzalez De La Fuente et al., 1995; Guibert et al., 1996). Once the sarcoplasmic reticulum was depleted in calcium, extracellular calcium was reintroduced in the incubation medium allowing the activation of store-operated calcium permeable channels in the plasma membrane. The role of extracellular calcium was studied by incubating IPA in calcium free KHB solution containing 0.4 mM EGTA. Unlike the absence of calcium from sarcoplasmic reticulum, absence of extracellular calcium significantly reduced 5-HT-induced ${{\text{O}}_{2}}^{\underline{•}}$ increase (*n* = 12–13) (Figure 3A). Moreover, absence of both intra and extracellular calcium did not further affect 5-HT-induced ${{\text{O}}_{2}}^{\underline{•}}$ production (*n* = 7) (Figure 3A).

We have then investigated the role of the voltage-dependent and voltage-independent calcium channels on 5-HT-induced ${{\text{O}}_{2}}^{\underline{•}}$ increase by using their respective blockers in rat IPA. Nicardipine (1 μM), an L- type voltage-dependent calcium channels blocker, did not prevent ${{\text{O}}_{2}}^{\underline{•}}$ increase in response to 5-HT (Figure 3B). LOE-908 (10 μM), a voltage-independent calcium channel inhibitor and RHC-80267 (50 μM), a DAG lipase inhibitor which will prevent arachidonic acid to stimulate voltage-independent calcium channels, were then used because they were already known to decrease contraction and calcium response to 5-HT in rat IPA (Guibert et al., 2004). LOE 908 and RHC-80267 both significantly decreased 5-HT-induced ${{\text{O}}_{2}}^{\underline{•}}$ increase (Figure 3C). Altogether, 5-HT stimulation of rat IPA produced ${{\text{O}}_{2}}^{\underline{•}}$ via the activation of the 5-HT_2_ receptors and a calcium influx through voltage-independent calcium channels.

### Role of ROS sources in the production of ${{\text{O}}_{2}}^{\underline{•}}$ by 5-HT in rat IPA

**Figure 4:**
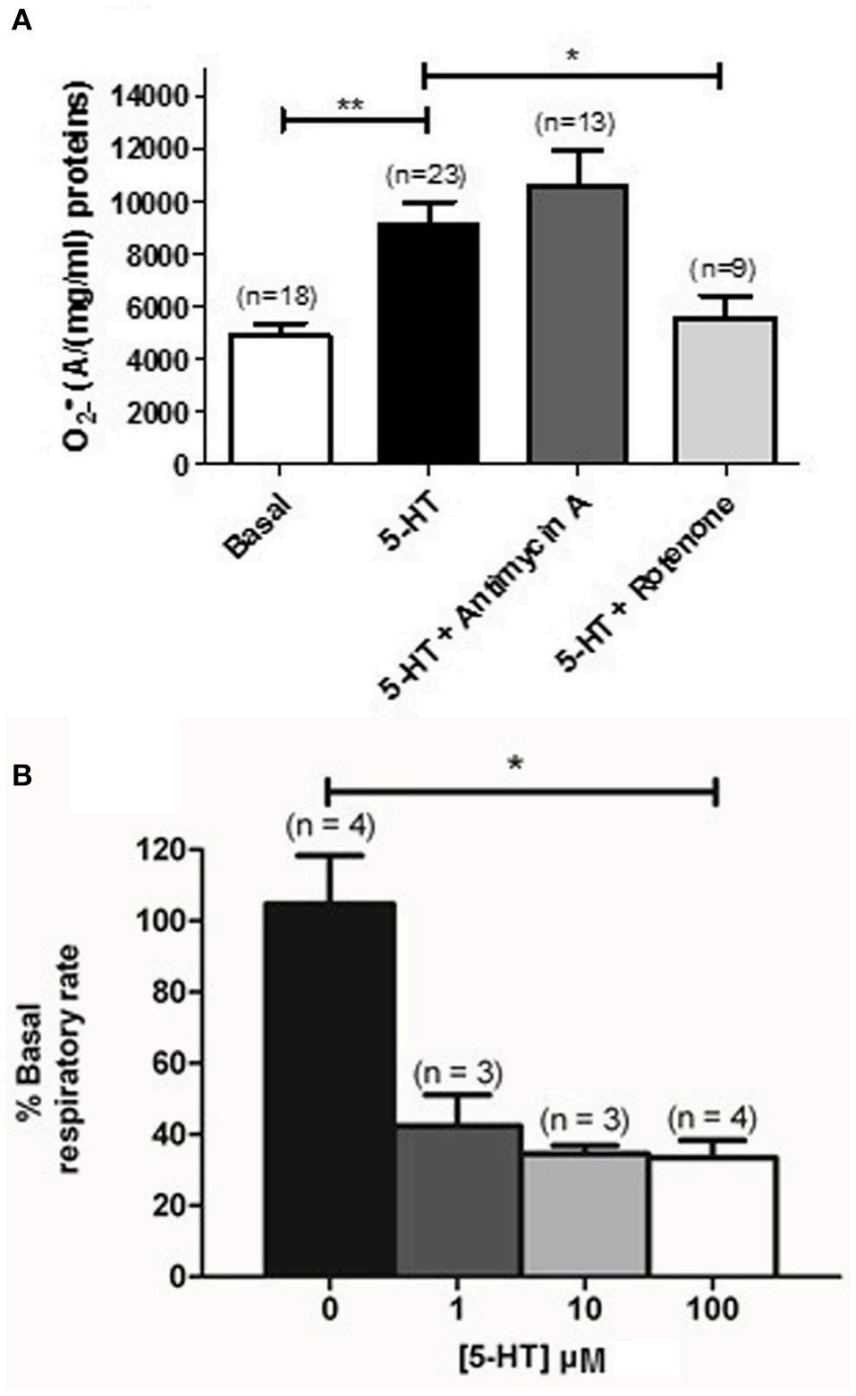
**Mitochondrial activity is modified in response to 5-HT**. **(A)** Effect of inhibitors of the complex III and I of the mitochondrial respiratory chain (Antimycin A, 10 μM and rotenone, 100 nM, dark and light gray columns respectively) is shown on the ${{\text{O}}_{2}}^{\underline{•}}$ produced by 100 μM 5-HT in rat IPA. n is the number of vessels. **(B)** Illustrates mitochondrial activity, expressed as a % of basal respiratory rate in response to cumulative concentrations of 5-HT (1 to 100 μM) in PASMC. n is the number of rats. Data are expressed as mean ± SEM. ^**^ and ^*^ mean a significant difference for *P* < 0.01 and *P* < 0.05 respectively.

**Figure 5:**
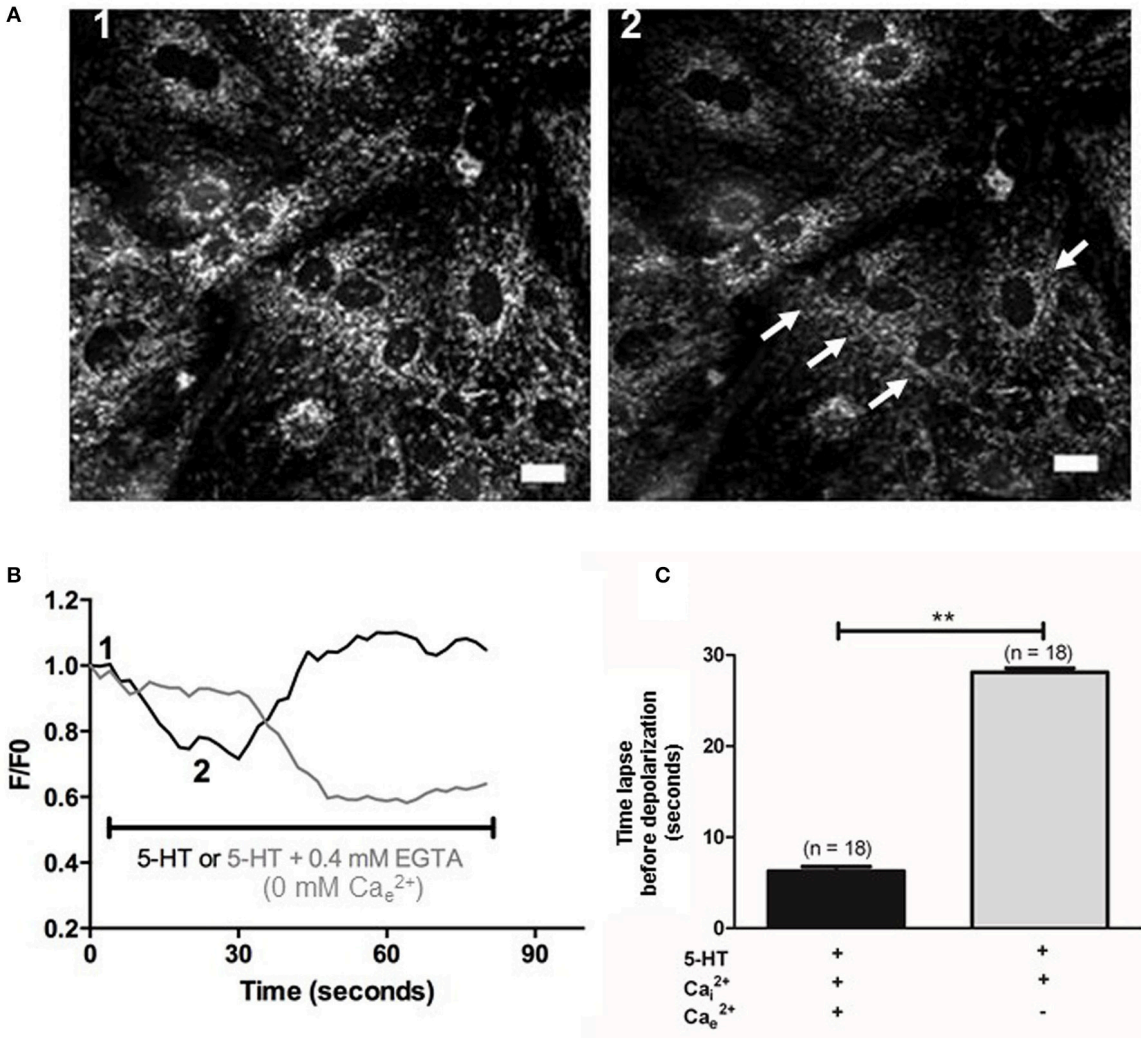
**Effect of 5-HT on mitochondrial membrane potential in PASMC. (A)** Images 1 and 2 represent mitochondrial fluorescence before and after application of 5-HT (100 μM). White arrows indicate cells with the highest decrease in fluorescence following 5-HT stimulation. Scale bars are 20 μm. **(B)** Typical traces of mitochondrial membrane potential in response to 5-HT in the presence of 2 mM ${\text{Ca}}_{\text{e}}^{2+}$ (black line) or in the absence of ${\text{Ca}}_{\text{e}}^{2+}$ and the presence of 0.4 mM EGTA (light gray line). One and two indicate the F/F0 value for one cell from the corresponding images 1 and 2 shown in **(A)**. F/Fo is the ratio of the fluorescence values F over the basal fluorescence F0 (before application of the agonists). **(C)** Values of the time lapse between bath-application of 5-HT and the beginning of the mitochondrial membrane potential variation in the presence and in the absence of extracellular Ca^2+^ (black and light gray columns respectively). Data are expressed as mean ± SEM. ^**^means a significant difference for *P* < 0.01. n is the number of cells tested.

METC is known to be an important source of ${{\text{O}}_{2}}^{\underline{•}}$ (Perez-Vizcaino et al., 2010). Using EPR recordings in rat IPA, we showed that rotenone (100 nM), an inhibitor of the complex I of the METC significantly decreased the amount of ${{\text{O}}_{2}}^{\underline{•}}$ produced by 5-HT (100 μM) (Figure 4A). However, antimycin A (10 μM), a complex III cytochrome b_H_ inhibitor did not exhibit any significant effect on 5-HT-induced ${{\text{O}}_{2}}^{\underline{•}}$ production in rat IPA (Figure 4A). Rotenone did not modify basal ${{\text{O}}_{2}}^{\underline{•}}$ level (*n* = 6—data not shown). To confirm the role of mitochondria, we addressed the effect of 5-HT on the mitochondrial respiratory rate and on the mitochondrial membrane potential. 5-HT significantly decreased the mitochondrial respiratory rate (Figure 4B). The mitochondrial membrane was transiently depolarized in response to 5-HT 100 μM in the presence of extracellular calcium (Figures 5A,B) whereas, in its absence, the depolarization was strongly delayed and sustained (Figures 5B,C).

**Figure 6:**
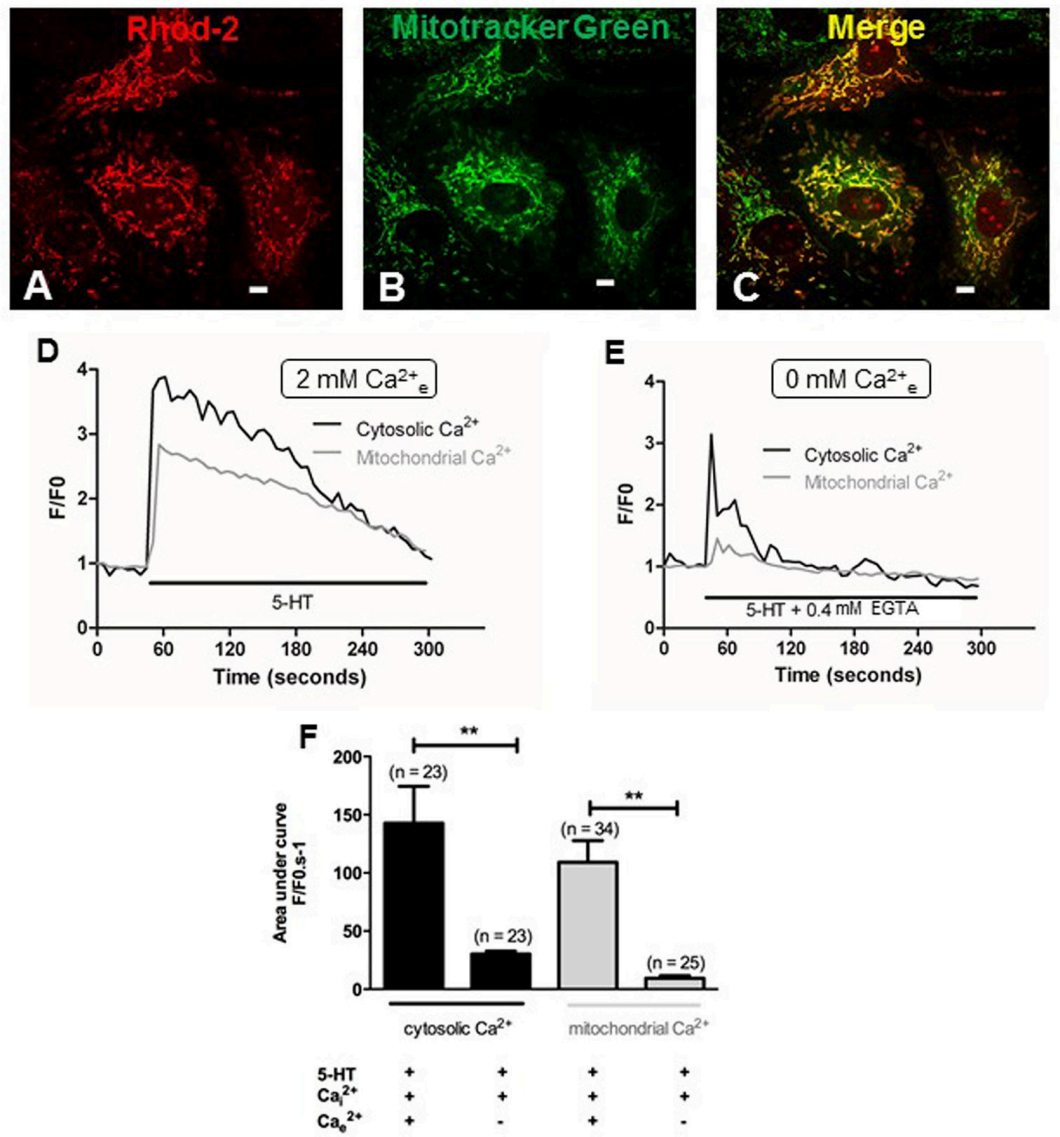
**Role of extracellular calcium in the cytosolic and mitochondrial calcium signals induced by 5-HT. (A,B)** Show PASMC loaded with rhod-2 (1 μM) and mitotracker green (400 nM) respectively. **(C)** Is the merge of **(A,B)**, illustrating colocalization of rhod-2 and mitotracker green as an index of the mitochondrial network in yellow. Scale bars are 20 μm. Typical traces of simultaneous recordings of cytosolic (black lines) and mitochondrial (light gray lines) Ca^2+^ levels in response to 5-HT (100 μM) in PASMC are shown in the presence or in the absence of extracellular Ca^2+^ (**D** and **E** respectively). **(F)** Histograms of cytosolic and mitochondrial calcium variations (black and light gray columns respectively) induced by 5-HT (100 μM) in the presence and in the absence of extracellular Ca^2+^. n is the number of cells. ^**^means a significant difference for *P* < 0.01.

**Figure 7:**
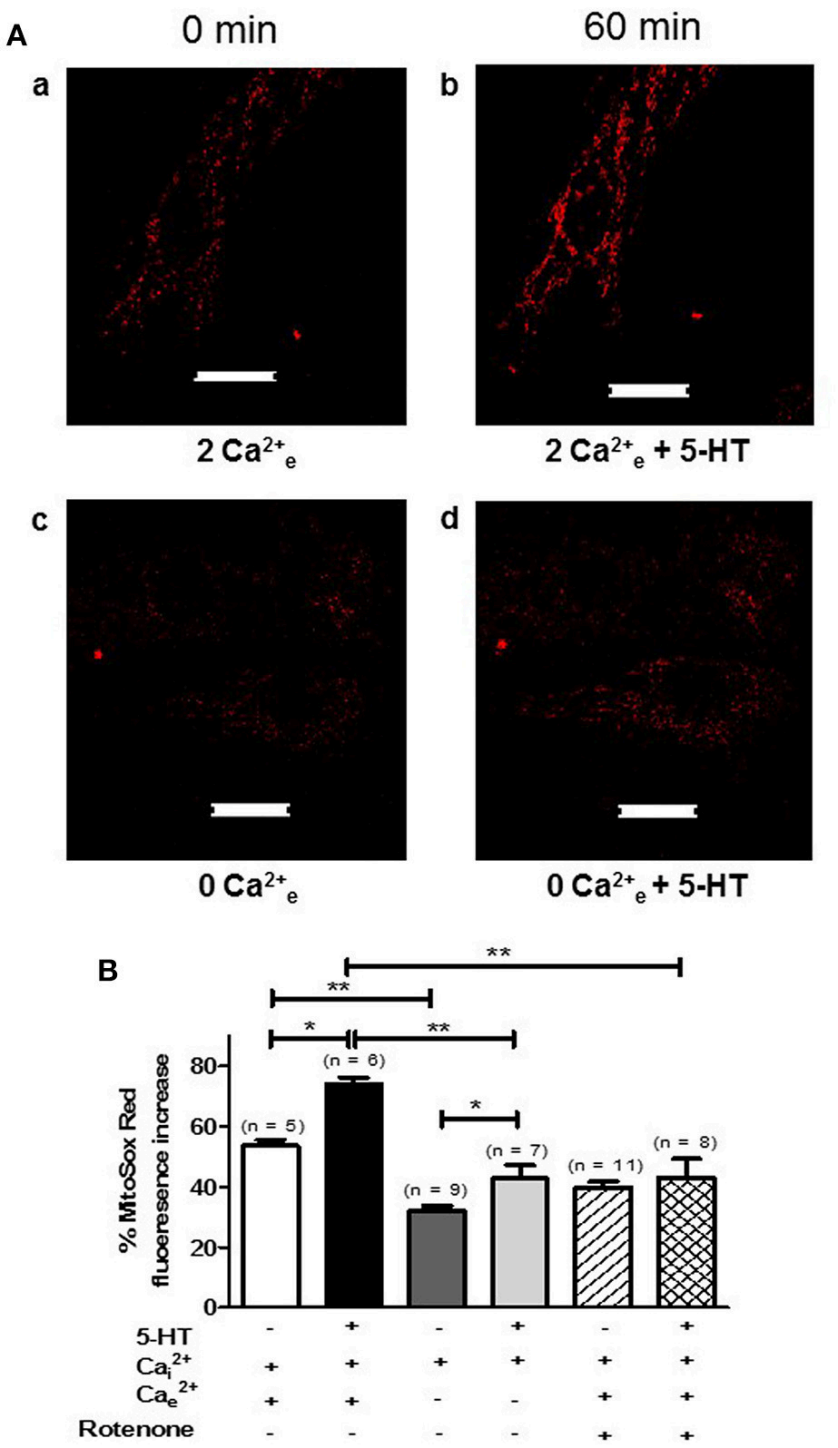
**5-HT-induced mitochondrial ${{\text{O}}_{2}}^{\underline{•}}$ production is sensitive to extracellular calcium and rotenone in rat PASMC. (A)** Shows images of mitochondrial mitosox fluorescence in untreated conditions (in presence or absence of extracellular calcium, **Aa** and **Ac** respectively) and following a 60 min incubation with 5-HT (100 μM) in the presence or absence of extracellular calcium (**Ab** and **Ad** respectively). Scale bar is 20 μm for all images. **(B)** Means ± SEM of the percent change in mitosox fluorescence after 60 min in untreated conditions in the presence of extracellular calcium or not (white and dark gray columns respectively), following 60 min of 5-HT (100 μM) incubation in the presence of extracellular calcium or not (black and light gray columns respectively), in unstimulated conditions with rotenone (100 nM) (hatched column) and finally following a 60 min incubation with 5-HT, rotenone and both intra and extracellular calcium (double hatched column). n is the number of cells tested. ^*^ and ^**^ indicate a significant difference for *P* < 0.05 and *P* < 0.01 respectively.

Since extracellular calcium influx and mitochondria both play a key role in the production of ${{\text{O}}_{2}}^{\underline{•}}$ by 5-HT (Figures 3A,C, 4A), we hypothesized that calcium influx induced by 5-HT would trigger an increase in mitochondrial calcium. We have thus simultaneously measured cytosolic and mitochondrial calcium levels in PASMC, in response to 5-HT in the presence and in the absence of extracellular calcium. Fluorescent labeling of PASMC with mitotracker green, a mitochondrial marker, and rhod-2, a mitochondrial calcium dye, shows a high level of colocalization demonstrating the specific targeting of rhod-2 to the mitochondrial network (Figures 6A–C). 5-HT (100 μM) increased both cytosolic and mitochondrial calcium levels in the presence of extracellular calcium (Figures 6D,F). In the absence of 5-HT stimulation, both cytosolic and mitochondrial calcium levels did not vary over time (data not shown). In the absence of extracellular calcium, the rise in cytosolic calcium in response to 5-HT was significantly decreased and the rise in mitochondrial calcium almost disappeared (Figures 6E,F). It should be noted that mitochondrial calcium increase is slightly delayed compared to cytosolic calcium increase (Figure 6D) suggesting that calcium influx may trigger the increase in mitochondrial calcium. In addition, we specifically measured mitochondrial ${{\text{O}}_{2}}^{\underline{•}}$ with the fluorescent probe mitosox. 5-HT (100 μM) significantly increased mitochondrial ${{\text{O}}_{2}}^{\underline{•}}$ levels (Figures 7Aa,Ab,B) and this ${{\text{O}}_{2}}^{\underline{•}}$ increase was significantly inhibited in the absence of extracellular calcium (Figures 7Ac,Ad,B) or the presence of rotenone (100 nM) (Figure 7B). Altogether, these results confirm the role of mitochondria, mitochondrial complex I and extracellular calcium in the signaling pathways involved in response to 5-HT in rat IPA.

## Discussion

Our findings provide the first comprehensive description of the role of mitochondria and calcium in 5-HT-induced ${{\text{O}}_{2}}^{\underline{•}}$ increase in rat IPA. Here, we clearly show that 5-HT acts on 5-HT_2_ receptors to activate a calcium influx responsible for mitochondrial calcium increase ultimately leading to a mitochondrial ${{\text{O}}_{2}}^{\underline{•}}$ production.

In the present study, we first confirmed that 5-HT produces ${{\text{O}}_{2}}^{\underline{•}}$ by means of: (i) EPR recordings and, (ii) measurement of ONOO^−^ which, indirectly, quantifies ${{\text{O}}_{2}}^{\underline{•}}$. Interestingly, the production of ONOO^−^ was consistent with our previous study, in which we demonstrated that ${{\text{O}}_{2}}^{\underline{•}}$ negatively controls endothelial-induced relaxation by reducing cGMP production, likely due to scavenging of the endothelial NO (Billaud et al., 2009).

The present data demonstrated that 5-HT-increased ${{\text{O}}_{2}}^{\underline{•}}$ levels is sensitive to inhibition of 5-HT_2_ receptors with ketanserin, to extracellular calcium removal and to inhibition of voltage-independent calcium channels with RHC-80267 and LOE-908 (Figures 2, 3). On the other hand, we previously demonstrated that the contraction to 5-HT is (i) sensitive to the same blockers (ketanserin, calcium free solution, RHC-80267 and LOE 908) (Guibert et al., 2004; Rodat et al., 2007; Rodat-Despoix et al., 2008) and (ii) implicates ${{\text{O}}_{2}}^{\underline{•}}$ produced by 5-HT (Billaud et al., 2009), thus confirming that 5-HT-induced ${{\text{O}}_{2}}^{\underline{•}}$ production seems to be involved in the contraction to 5-HT under physiological conditions in rat IPA. Consistently, other groups have also demonstrated that the contraction to 5-HT is dependent on stimulation of 5-HT_2A_ receptors and extracellular calcium in rat pulmonary artery (MacLean et al., 1996; Yuan et al., 1997).

The 5-HT concentration used in the present study is high, much higher than the 5-HT levels detected in physiological conditions (around 10 nM) (Kéreveur et al., 2000). However, 10 nM is the plasma 5-HT levels and we do not really know what is the actual concentration at the site of the pulmonary arteries and, more specifically, at the site of the smooth muscle cells. Taking into account that 5-HT is also produced in the lung by pulmonary neuroendocrine cells, endothelial cells and neuroepithelial bodies distributed throughout the airways (see introduction), we can speculate that smooth muscle cells are in contact with a higher 5-HT concentration than the circulating 5-HT levels. Moreover, the ROS-dependent process, described here, is supposed to be involved in the contractile response to 5-HT in rat IPA (Billaud et al., 2009) and we previously showed that the maximal contraction to 5-HT was obtained for a concentration of 100 μM. For a 5-HT concentration of 1 μM, the amplitude of the contraction in rat IPA was of only about 10–20% of that induced by a high potassium solution (80 mM) or about 30% of the maximal contraction (Guibert et al., 2004, 2005; Rodat et al., 2007; Rodat-Despoix et al., 2008; Billaud et al., 2009). Moreover, at lower concentration, the percentage of responding isolated cells to agonists is usually also lower, especially regarding calcium signal. Altogether, we thus decided to use a high concentration of 5-HT i.e., 100 μM.

Extensive studies have been performed on ROS production and on the sources involved in such production in the pulmonary circulation, especially in the case of hypoxic pulmonary vasoconstriction or pulmonary hypertension (Archer et al., 1993; Leach et al., 2001; Rathore et al., 2008; Dromparis and Michelakis, 2013) but only one study addressed the issue of ROS production in response to vasocontractile agonist stimulation (Liu and Folz, 2004). Liu and Folz (2004) have shown the role of the NADPH oxidase in the production of ${{\text{O}}_{2}}^{\underline{•}}$ by 5-HT in mice PA by using pharmacological and genetic approaches (apocynin and gp91^phox^ knock-out mice, respectively) but they did not address the role of mitochondria. Other studies have shown, in bovine PASMC, that 5-HT is up taken by the 5-HT transporter and produces ${{\text{O}}_{2}}^{\underline{•}}$ which is then dismutated by SOD into H_2_O_2_ and participates to the 5-HT mitogenic activity (Lee et al., 1998, 2001). 5-HT_1_ and 5-HT_2_ agonists fail to enhance ${{\text{O}}_{2}}^{\underline{•}}$ release in bovine PASMC (Lee et al., 2001). In those studies, the role of ${{\text{O}}_{2}}^{\underline{•}}$ and/or H_2_O_2_ in the contraction to 5-HT has not been addressed. Interestingly, in our case, not only 5-HT_2_ receptors activation increases ${{\text{O}}_{2}}^{\underline{•}}$ levels but citalopram, a 5-HT transporter inhibitor, does not affect 5-HT-induced ${{\text{O}}_{2}}^{\underline{•}}$ production (Figure 2A) therefore we believe that we demonstrated the presence of another 5-HT signaling pathway associated to ${{\text{O}}_{2}}^{\underline{•}}$ production in rat IPA. Moreover, since the main role of the pulmonary circulation is to control blood/oxygen supply to the organs, hypoxic pulmonary vasoconstriction occurs in case of reduced alveolar oxygen, in order to redirect blood to more ventilated areas of the lung and ensure optimal gas exchange. Hypoxic sensor is still not clear however hypoxic pulmonary vasoconstriction is known to depend on mitochondrial-derived ROS production which reduces voltage-dependent potassium channels current and thus, increases intracellular calcium level in smooth muscle (Archer et al., 1993; Yuan et al., 1993; Leach et al., 2001; Dromparis and Michelakis, 2013; Freund-Michel et al., 2014). Acute hypoxia has also been shown to induce 5-HT secretion in pulmonary neuroendocrine cells (Cutz et al., 1993). Therefore, from our present results, we can speculate that the mitochondrial ROS involved in hypoxic pulmonary vasoconstriction may be produced by 5-HT coming from pulmonary neuroendocrine cells.

Under pathophysiological conditions, NO reduction by ${{\text{O}}_{2}}^{\underline{•}}$ has already been observed in systems that overproduce ROS such as pulmonary and systemic hypertension (Brennan et al., 2003; Ago et al., 2011). However, so far, no study has demonstrated any role of mitochondrial ${{\text{O}}_{2}}^{\underline{•}}$ in the vascular responses to 5-HT or any other vasoconstrictor in physiological conditions. We have several lines of evidence arguing in favor of a role of mitochondria in response to 5-HT in general and, more specifically, in the production of ${{\text{O}}_{2}}^{\underline{•}}$ by 5-HT under physiological conditions. First of all, 5-HT significantly decreased mitochondrial respiratory rate (Figure 4B) and significantly depolarized mitochondrial membrane (Figure 5). Moreover, ${{\text{O}}_{2}}^{\underline{•}}$ produced by 5-HT is blocked by rotenone (Figure 4A) and 5-HT increases both the mitochondrial calcium concentration and ${{\text{O}}_{2}}^{\underline{•}}$ levels (Figures 6D,F, 7, respectively). Interestingly, in the absence of extracellular calcium, the increase in mitochondrial calcium concentration is almost suppressed (Figures 6E,F) and change in mitochondrial membrane potential is strongly delayed (Figures 5B,C) which is in accordance with data from the literature demonstrating that calcium influxes are buffered by mitochondria positioned close to the plasma membrane (McCarron et al., 2012). According to our results and findings from the literature, we thus suggest that extracellular calcium may be taken up by the mitochondria to depolarize mitochondrial membrane in order to produce ${{\text{O}}_{2}}^{\underline{•}}$.

In conclusion, 5-HT-induced production of ${{\text{O}}_{2}}^{\underline{•}}$ is mediated by 5-HT_2_ receptors and is dependent on the complex I of the METC. Extracellular calcium influx plays an important role in this process. Our study emphasized the presence of a mitochondrial calcium-dependent ${{\text{O}}_{2}}^{\underline{•}}$ production by 5-HT under physiological conditions thus revealing a novel unexpected physiological role for mitochondrial ${{\text{O}}_{2}}^{\underline{•}}$ in response to 5-HT. Since calcium and superoxide anion are involved in the pulmonary arterial contraction to 5-HT (Billaud et al., 2009), we thus described, in the present study, a potential alternative and novel mechanism for vascular tone regulation via the action of ${{\text{O}}_{2}}^{\underline{•}}$. Moreover, ${{\text{O}}_{2}}^{\underline{•}}$, 5-HT concentrations, intracellular calcium and vascular tone are known to be increased in pulmonary hypertension suggesting that such mitochondrial ${{\text{O}}_{2}}^{\underline{•}}$ produced by 5-HT may also be of great importance in case of pulmonary hypertension. Such hypotheses need to be further investigated. 5-HT, ROS, and peroxynitrites are also involved in various vascular pathologies. Thus, such better understanding of 5-HT-induced ROS production could be of potential interest for new therapeutic strategies regarding pulmonary hypertension and vascular pathologies in general.

## Author contributions

NG, MB, RR, MD, JG, BI, RM, J-PS, and CG conception and design of research; NG, CG, MB, MD, and JG performed experiments; NG and CG analyzed the data; NG, CG, MB, MD, and JG interpreted results of experiments; NG and CG prepared figures; NG, CG drafted manuscript; NG, MB, RR, MD, JG, BI, RM, J-PS, and CG edited and revised manuscript and approved final version of manuscript.

## Funding

This work was supported by Conseil Régional d'Aquitaine (20111302006) and INSERM.

### Conflict of interest statement

The authors declare that the research was conducted in the absence of any commercial or financial relationships that could be construed as a potential conflict of interest.

## Abbreviations

5-HT: serotonin
EPR: electron paramagnetic resonance
IPA: intrapulmonary artery
KHB: Krebs-Hepes-Bicarbonate
METC: mitochondrial electron transport chain
${{\text{O}}_{2}}^{\underline{•}}$: superoxide anion
ONOO^−^: peroxynitrite
PA: pulmonary artery
PASMC: pulmonary artery smooth muscle cells
ROS: reactive oxygen species
SOD: superoxide dismutase.
